# Augmentation of learning in schizophrenia by D-serine is related to auditory and frontally-generated biomarkers: A randomized, double-blind, placebo-controlled study

**DOI:** 10.21203/rs.3.rs-2943290/v1

**Published:** 2023-05-19

**Authors:** Viraj Govani, Adithya Shastry, Daniel Iosifescu, Preetika Govil, Megan Mayer, Tarek Sobeih, Tse Choo, Melanie Wall, Pejman Sehatpour, Joshua Kantrowitz

**Affiliations:** Nathan Kline Institute; Department of Psychiatry, Columbia University

**Keywords:** NMDA receptor, schizophrenia, target engagement, auditory learning, oscillations, Beta power

## Abstract

Auditory cognition is impaired in schizophrenia, and typically engages a complex, distributed, hierarchical network, including both auditory and frontal input. We recently demonstrated proof of principle for the target engagement of an N-methyl-D-aspartate-type glutamate receptor (NMDAR) agonist + auditory targeted remediation (d-serine+AudRem) combination, showing significant improvement in auditory-learning induced plasticity and mismatch negativity. In this secondary analysis, we report on frontal EEG outcomes, assessing for both generalized effects and the mechanism of auditory plasticity.

21 schizophrenia or schizoaffective disorder participants were randomized to three 1x weekly AudRem + double-blind d-serine (100 mg/kg) visits. In AudRem, participants indicated which paired tone was higher in pitch. The focus of this secondary analysis was a frontally (premotor) mediated EEG outcome— event-related desynchronization in the b band (b-ERD), which was shown to be sensitive to AudRem in previous studies.

d-S erine+AudRem led to significant improvement in b-ERD power across the retention and motor preparation intervals (F_1,18_=6.0, p=0.025) vs. AudRem alone. b-ERD was significantly related to baseline cognition, but not auditory-learning induced plasticity.

The principal finding of this prespecified secondary analysis are that in addition to improving auditory based biomarkers, the d-serine+AudRem combination led to significant improvement in biomarkers thought to represent frontally mediated dysfunction, suggesting potential generalization of effects. Changes in auditory-learning induced plasticity were independent of these frontally mediated biomarkers. Ongoing work will assess whether d-serine+AudRem is suffi cient to remediate cognition or whether targeting frontal NMDAR deficits with higher-level remediation may also be required.

**Trial Registration:**

NCT03711500

## Introduction

In addition to cognitive deficits in attention, working memory and executive functioning [1], schizophrenia is associated with functionally relevant early auditory processing deficits (EAP) [2–4], exemplified by deficits in distinguishing differences in pitch (frequency) between successive tones (e.g., tone matching thresholds (TMT)) [5]. Schizophrenia patients require an approximately a 20% difference in pitch (%Δf) to differentiate two tones separated by a brief (300 ms) delay to match performance at 5% Δf in healthy volunteers [6]. EAP deficits contribute to deficits in more complex general and auditory cognition [7], important for both occupational [8, 9] and social functioning [10–15]. EAP deficits also contribute to neuroplastic deficits in both learning auditory information [16, 17] and in benefitting from various forms of cognitive remediation [18–20], including auditory cortex targeted remediation (AudRem). The auditory cortex is the center of a complex, distributed, hierarchical network [21] that integrates the representation of auditory stimuli and frontal input. EAP deficits appear to be N-methyl-D-aspartate-type glutamate receptor (NMDAR)-dependent, as TMT depends on the formation of a NMDAR-dependent ‘echoic’ memory trace [4].

Across studies [17, 22–31], we and others demonstrated proof of principle for several auditory based measures, including mismatch negativity (MMN), θ and behavioral plasticity as pharmacodynamic “target engagement” biomarkers for both glutamatergic based compounds alone and in combination with AudRem. We have studied an AudRem that is a dynamic version of TMT, in which participants are presented with two paired tones (e.g., Stimulus 1 (“reference”) and Stimulus 2 (“test”): S1 and S2) and indicate which tone is higher in pitch (frequency, Hz). Most recently [22], we saw a significant effect for sustained plasticity improvement and a significantly larger MMN for a d-serine + AudRem compared to AudRem alone.

Previous studies in preclinical [32–35], healthy [36, 37] and dyslexic [38, 39] cohorts have suggested that in most populations, the auditory-learning induced plasticity generated by AudRem is mediated by the prefrontal cortex [40–42] forming a perceptual anchor to the stimulus regularity of the reference tone generated in auditory cortex [43]. By contrast, in schizophrenia, auditory plasticity deficits appear to be mediated by primary auditory cortical level deficits. Isolated bilateral auditory cortical lesions [44–47] lead to a dramatic worsening in pitch TMT. However, schizophrenia patients do not show increased TMT deficit when distracting information is included during TMT testing [48, 49]. Thus, impairing frontally mediated attention does not impair TMT, arguing for primarily auditory cortical mediation of TMT deficits in schizophrenia and against primarily frontal mediation of auditory cortical deficits. As opposed to steady-state EAP, the pattern of impairment or “bottlenecks” for plastic EAP deficits in Sz is understudied.

We [22, 23] and others [38, 39, 41] have previously reported that AudRem produces a characteristic EEG response. In event or stimulus related response analysis (ERP) of AudRem, there is a N1–P2 response complex for each of the two tones (reference and test) that indexes activation at the level of sensory (auditory) [50] and dorsal attention regions [51]. In AudRem studies of dyslexia, the N1 is unaffected, and deficits were seen only in P2 [41], consistent with frontally driven deficits [52]. In dyslexia and healthy controls, the P2 was predictive of performance [41, 53]. The P2 is understudied in schizophrenia but has been localized to the parietal cortex [51] and may be augmented (enhanced) in a two-tone pair paradigm like AudRem, potentially due to a failure to attention [54].

Electrophysiological activity is divided conventionally into discrete bands which reflect differential underlying local-circuit processes/oscillations [55–58], thus complementing ERP by assessing circuit level function and reflecting alterations in single-trial power ([e.g. 57,59]). The oscillation response of AudRem [23] consists of 3 main components: 1) sensory responses immediately following the S1 (0–200 ms) and S2 (1000–1200 ms) stimuli; 2) an information ‘retention interval” response that occurs between the S1 and S2 stimuli (400–1000 ms), and 3) a “motor-preparation” interval that occurred following the S2 response (1200–1500 ms).

ERP responses, including mismatch negativity (MMN) and N1, are typically associated with increases in θ (4–7 Hz) power [60] during the sensory response, and thus are expected to be related to auditory sensory nodes/networks. As recently reviewed [61, 62], event-related desynchronization in the β band (β-ERD) appears crucial for cognition and motor planning. Specifically, suppression of ongoing β (12–30 Hz) activity (e.g., ERD in the β band) has been tied to bringing regions “on-line” during cognitive processing, e.g., frontoparietal control (β [63, 64]) and localized to premotor, dorsal-lateral frontal and parietal regions [65]. Since ERD activity is not tightly time-locked to the eliciting stimulus, it is poorly represented within the standard ERP response.

Based on our previous work and prespecified hypothesis [22, 66], for this secondary analysis, we focus on this frontally generated biomarker—β-ERD and its relationship with auditory plasticity and cognition, working memory, attention and frontoparietal control shown to be sensitive to AudRem [23, 64].

## Patients and Methods

### Participants:

This was a parallel-group, randomized, placebo-controlled, double-blind investigation conducted at New York State Psychiatric Institute (NYSPI) [67] and the Nathan Kline Institute (NKI) between March 2019 and April 2021. The study was approved by our IRB, with written informed consent obtained prior to participation.

Criteria were modified from consensus criteria for cognition studies [68]. Participants were medically-stable, aged 18–50 with schizophrenia or schizoaffective disorder and impaired auditory and overall cognition [MCCB composite and verbal memory domain at least 0.5 standard deviations below the mean (T-score ≤ 45) and/or a tone-matching-threshold (TMT) ≤ 77.7% [69]], on a stable antipsychotic dose for ≥ 4 weeks, had normal kidney function with an estimated Glomerular Filtration Rate ≥ 60 and had moderate or lower cognitive disorganization (Positive and Negative Symptom Scale [70] (PANSS) P2 ≤ 4) to ensure ability to focus on the task.

## Design

Overall, 45 participants were randomized to three 1x weekly AudRem. For this secondary analysis, we report on participants randomized to double-blind d-serine 100 mg/kg (12 total) or placebo (9 total), which was the optimal dose in the main study [22]. During study visits, pre-treatment MMN was collected (Visit 1 only), followed by AudRem EEG beginning 30–45 minutes after study drug administration to allow for training during peak d-serine levels [25, 71], immediately followed by post-treatment MMN (Visit 2 and 3 only) and safety labs. Randomization lists were produced by the study biostatistician. Randomization was stratified by baseline TMT [69].

### AudRem program:

We utilized an AudRem program that was initially developed and validated in developmental dyslexia [72] and modified from our published work [23] to enhance learning by using the same reference tone throughout visits and carrying forward the visit end pitch threshold to the next session.

In AudRem, participants were presented with paired tones (e.g., Stimulus 1 (“reference”) and Stimulus 2 (“test”): S1 and S2) and indicate which tone is higher in pitch (frequency, Hz). After a practice block of 10 pairs to ensure ability to comprehend test instructions, participants completed 240 pairs per visit. Tones were presented at a comfortable volume and were 50 ms in duration with a stimulus onset asynchrony (SOA) of 1000 ms. The intertrial interval was 1800 ms. All tones were presented binaurally with the apparent location in the center midline. In the first pair, the between tone ratio was 50% (e.g. 1000 ± 500 Hz), and the diffi culty level was adjusted to maintain a steady (~ 70% correct) level of performance with a four-up/one-down staircase procedure. The smallest possible tone pair ratio was 1%. The same reference tone was used for all sessions (1000 Hz) and began the next treatment session at the same pitch ratio that the previous session ended at. Ratios in each pair were averaged across 10-trial pairs.

As previously [23], plasticity was defined as improved (smaller) pitch thresholds between paired auditory stimuli after AudRem, (%Δf S1/S2), operationalized as **plasticity improvement:** change in pitch threshold [(test tone Hz subtracted from reference tone Hz) divided by the reference tone Hz] between the initial plateau (mean of trials 20–30 of treatment visit 1) and the pitch threshold end of treatment visit 1, 2 or 3.

## Baseline Measures

Auditory cognition was assessed by the Verbal Memory domain of the MCCB, EAP by the TMT [73], along with the full MCCB and ancillary auditory cognition measures (Table 1).

### EEG methods:

Continuous EEG data were acquired through Brain Vision Brainamp MR Plus amplifier system using 64 scalp electrodes (10–10 system), impedances < 5 kΩ, referenced to the FCz electrode, bandpass filtered from 0.05 to 100Hz, and digitized at 500 Hz. ERP analysis was performed offi ine using MATLAB (R2020b, Mathworks, Natick, MA) and EEGLAB, ERPLAB toolboxes. Data were examined for eye, facial muscle, and residual artifacts, which were removed using independent components analysis (EEGLAB 2020.0, Delorme and Makeig). A 0.1–80 Hz filter was applied, along with a 60 Hz line noise filter, and the electrode information was re-referenced to the average linked mastoids. Trials with amplitudes that exceeded 120 mV were removed. Occasional noisy channels were substituted by interpolated data from neighboring channels. AudRem EEG used previously described methodology [22, 23] and latency windows.

### Event-related potential analysis (AudRem):

Epochs were defined from − 500 ms to 2000 ms. Responses were averaged over four electrodes: Fz, FC1, FC2, Cz. Data were high-pass filtered at 0.1 Hz and low-pass filtered at 30 Hz. ERPs were obtained by time locking on to the onset of all stimuli and averaging across trials baselined from − 500 to 0 ms. Mean and peak amplitudes were calculated centered around the average peaks for all subjects for all three treatments for N1 to S1 (90–170 ms), P2 to S1 (195–285 ms), CNV (800–1000 ms, mean only), N1 to S2 (1080–1180 ms), and P2 to S2 (1180–1270 ms).

### Time-frequency Analysis (AudRem):

Time-frequency analysis was performed using MATLAB (R2020b, Mathworks, Natick, MA) and custom written scripts, and focused on four time windows. Two sensory response periods (0–200 ms post-S1 and S2), were meant to capture the initial sensory volley of activity, corresponding to the N1 response; a retention interval between S1 and S2 (400–1000 ms) was meant to capture the encoding activity; and a motor-preparation interval (200–500 ms post-S2) captured the activity in preparation of a response. The frequency ranges of interest were derived by averaging the TF data within each frequency range as θ (4–7Hz) and β (12–30Hz). Single-trial TF transformations were derived to compute baseline-corrected single-trial power and inter-trial coherence (ITC). ITC reflects the alignment in phase of spectral response across repeated trials ranging from 0 (no alignment) to 1 (perfect alignment). In general, changes in ITC in the absence of alterations in spectral power are thought to reflect stimulus induced phase reset of ongoing oscillatory activity. A frontocentral scalp ROI was derived by averaging the signal from electrodes Fz, FC1, FC2, and Cz.

### Statistical analysis:

Full analysis details were previously published [22]. For analyses described here, d-serine subjects are restricted to those randomized to 100mg/kg, the optimal dose in the main study. As previously [23], we used repeated-measures generalized linear models to assess β-ERD power across the retention and motor preparation intervals. Additionally, to test for treatment differences at each of the 3 study visits, two sample t-tests were performed for each outcome and for each interval and visit separately. Cohen’s d for between-group differences was computed as (outcome mean for the d-serine group minus mean for the placebo group) divided by the standard deviation of the outcome in the placebo group. As a sensitivity analysis, to account for skew in the β-ERD power distributions, tests were re-run after winsorizing the outcomes at the 95th percentile, yielding the same results for significance. Pearson correlations were used to assess functional relationships with plasticity and MCCB domains, and, due to skewed distributions, Spearman’s correlations were used to assess functional relationships with β-ERD power measures and MCCB domains. Correlations were then re-estimated partialling for treatment group. Values in the text are mean ± SD. Analyses were performed using SAS version 9.4.

## Results

### Sample:

The present report included a subsample 21 participants in the full study (**Supplemental Fig. 1**), receiving either d-serine 100 mg/kg (n = 12) or placebo (n = 9). There were no significant between-group differences in baseline characteristics (Table 1). 20 participants completed all three visits. One participant in the 100 mg/kg group was removed after visit 2 due to a parasite infestation at the participant’s apartment.

### Plasticity:

As previously reported [23], d-serine led to significant improvement within the 100 mg/kg treatment-group, showing statistically significant within group plasticity improvement in all three visits, demonstrating both acute (10.2±12.8%, 1st visit, d = 0.76) and sustained (11.1±13.1%, mean of 2nd and 3rd visits, d = 0.59) improvement. By contrast, placebo-treated participants did not show significant improvement after any visit (~ 5%, n.s.). There were no significant differences in between treatment-group testing vs. placebo.

### EEG:

**Figure 1:**
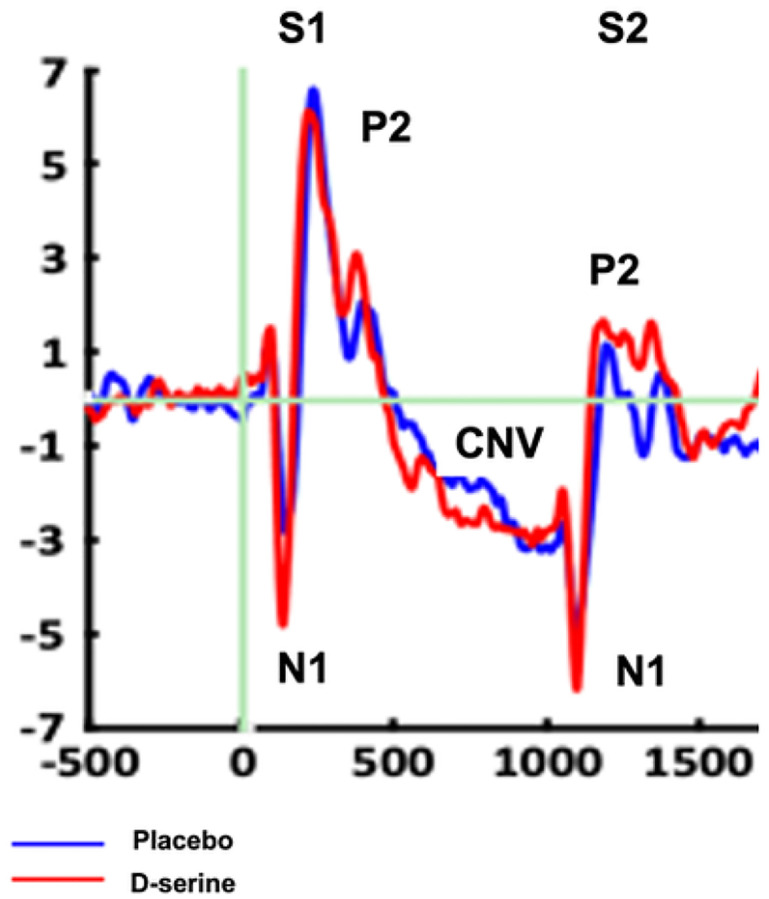
Grand average ERP waveforms for d-serine and placebo during plasticity session.

**Figure 2:**
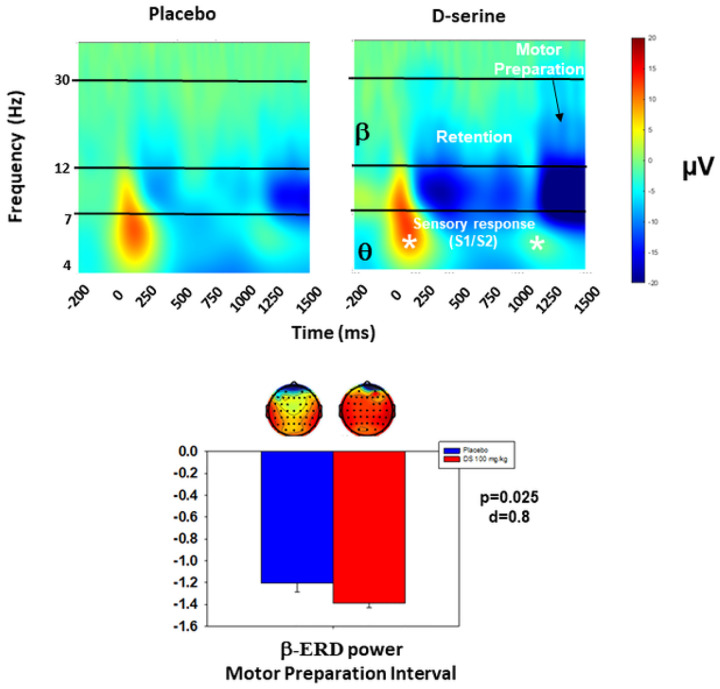
Top: Time-frequency plots for baseline corrected single-trial power. The timing of S1 and S2 presentation, along with b-ERD power during the retention and motor preparation interval are as illustrated. q, a and b ranges are illustarted. Bottom: Bar graph of b-ERD power during the motor preparation interval, Inset shows topographical plots (headmaps) for b-ERD power at 1340 ms, midpoint of the motor preparation interval.

As expected, ERP (Fig. 1) and oscillations (baseline-corrected single-trial power; Fig. 2) results were consistent with prior studies using auditory and visual paired stimulus-matching tasks [23, 38, 39, 41, 63, 64]. ERP showed the expected N1/P2 complex to both S1 and S2 stimuli and a CNV after S2. The oscillations response showed the expected sensory responses to S1 and S2 stimuli were characterized by increases in θ. During the retention and motor-preparation interval, activity was characterized primarily by a non-phase locked (“induced”) suppression of ongoing β activity (β-ERD). ITC patterns (not shown) were also consistent with previous studies.

As previously [23], a significant treatment effect was observed for β-ERD power across the retention and motor preparation intervals during the final visit (F_1,18_=6.0, p = 0.025, Fig. 2). Follow-up t-tests showed significantly larger (more negative) β-ERD for the d-serine treated participants for both the retention (t_18_ = 2.2, p = 0.041, d = 0.78) and motor preparation (t_18_ = 2.2, p = 0.038, d = 0.81) intervals at the final visit. Between group differences were not individually significant for β-ERD acutely (1st visit) for either the retention (t_19_ = 0.7, p = 0.49, d = 0.19) or motor preparation (t_19_ = 1.4, p = 0.18, d = 0.31) interval. No other significant ERP or oscillations changes were seen, and we did not replicate our previous finding of a significant treatment-group effect for θ-ITC during the motor interval.

### Relationship among measures:

As previously reported [22], a statistically significant correlation was seen between baseline auditory cognition and acute plasticity improvement (r = 0.46, p = 0.036), but not with general cognitive measures such as the MCCB composite, Working Memory or Attention and Vigilance domains (Table 2).

As expected, there were no significant correlations between plasticity improvement and β-ERD during the retention or motor preparation interval. However, a larger (more negative) β-ERD during both the retention and motor preparation interval at the final session was significantly related to higher baseline Attention and Vigilance scores and Working Memory scores (Table 2, **Supplemental Fig. 2**). In addition, β-ERD during the retention interval was additionally significantly correlated with Neurocognitive Composite (Table 2, **Supplemental Fig. 2**), and auditory cognition (r=−0.55, p = 0.012). All of these correlations remained significant when partialled for treatment group, with the exception of the association between β-ERD during motor preparation interval and working memory, which became trend-level (r=−0.45, p = 0.055).

## Discussion

Schizophrenia patients have deficits in auditory learning induced plasticity [16, 17] that may be rate limiting for improving overall cognition. The principal finding of this prespecified secondary analysis are that in addition to improving auditory based biomarkers (behavioral plasticity and MMN), the d-serine + AudRem combination led to significant improvement in biomarkers thought to represent frontally mediated dysfunction (β-ERD). These findings replicate our previous study [23] and suggest potential generalization of effects beyond improvement in auditory measures. Despite overall improvement, these frontally generated biomarkers were independent of plasticity improvement.

The present report was conducted as a target engagement study using Fast-Fail methodology [74–79], but our overall goal is to develop treatments for enhancing cognition in schizophrenia using a combined pharmacological and behavioral approach [16]. A fuller understanding of the mechanisms of auditory plasticity will assist in the development of target engagement biomarkers for early stage studies.

Cognition, including auditory cognition and the plastic auditory learning induced by AudRem, typically engages a complex, distributed, hierarchical network [21], including both auditory and frontal input. Our working hypothesis is that in schizophrenia, auditory cortical dysfunction is rate limiting for improvement in auditory plasticity and cognition. By assessing auditory and frontal EEG outcomes in Sz, we hope to refine the target for remediation programs, assessing whether AudCtx targeted remediation (AudRem) alone is suffi cient to remediate plasticity or whether targeting frontal NMDAR deficits with higher-level remediation may also be required, a crucial mechanistic and clinical question.

In our previous work [22, 23], we have shown that auditory plasticity is significantly related to both auditory cognition (MCCB verbal memory domain) and an auditory biomarker (MMN). Here, we have extended these findings beyond the auditory cortex, and replicated our prior findings [23] demonstrating that d-serine + AudRem combination led to significant enhancement of β-ERD across the retention and motor preparation intervals compared to AudRem alone. We also assessed auditory θ and N1, along with other prespecified EEG and oscillations outcomes, which did not show a significant d-serine effect. Thus, behavioral auditory plasticity (plasticity improvement), MMN and β-ERD have the most support as target engagement biomarkers for combined NMDAR agonist and AudRem treatment.

Consistent with our results, previous studies have implicated β-ERD in cognitive processing [80–85], particularly for motor planning [86, 87] and memory consolidation [85, 88]. In schizophrenia, β-ERD deficits may be related to poor psychosocial functioning and negative symptoms [89] and working memory [90]. As previously [23], β-ERD power did not significantly correlate with plasticity. β-ERD during the retention interval was predictive of baseline auditory cognition, while β-ERD during both the retention and motor preparation intervals was predictive of baseline higher order cognition. A prior study [91] has related β-ERD during the retention interval with working memory. Previous studies of visual memory have shown similar β-ERD changes during the motor preparation interval that correlated with cognitive measures among healthy volunteers [92] but not schizophrenia patients, consistent with our model that NMDAR-mediated auditory cortical deficits are rate-limiting.

Several limitations apply to this report. First, we note that while these exploratory analyses were prespecified, they were not corrected for multiple comparisons and should be interpreted cautiously. Second, we did not assess post-treatment cognition in this target-engagement study, and thus we cannot address whether any of these auditory or frontal biomarkers would predict post-treatment cognition. This limits our conclusions to correlations between putative biomarkers of auditory learning induced plasticity. We are thus unable to fully assess the link between auditory and frontally generated biomarkers and cognitive improvement. Our ongoing larger, longer study of the combination for clinically relevant outcomes will help address the utility of both auditory and frontal biomarkers as predictors of functionally relevant outcomes (NCT05046353).

## Figures and Tables

**Table 1:** Baseline demographics and cognition

	Placebo (n=9)	d-serine (n=12)	Test Statistic	p
**Age**	34.8±8	37.6±6.6	t_18_=0.9	0.39
**Male (%)**	77.8	50%	X^2^_1_=1.2	0.28
**Education (Years)**	13.6±4	13.0±2.1	t_18_=0.4	0.68
**CPZE (mg)**	1165±1181	604±447	t_18_=1.5	0.15
**Anticholinergic Cognitive Burden**	2.7±3.1	1.7±1.4	t_18_=1.2	0.23
**Auditory cognition (MCCB Verbal)**	34.3±5.5	37.2±7.6	t_18_=1.0	0.34
**TMT**	77.5±12.1	74.8±7.8	t_18_=0.7	0.47
**Neurocognitive Composite**	23.7±14.7	27.6±11.6	t_18_=0.7	0.50
**Attention and Vigilance**	32.4±13.4	32.1±10.3	t_18_=0.1	0.95
**Working Memory**	33±13.5	36.2±11.4	t_18_=0.6	0.58

**Table 2:** Relationship of Biomarkers with Baseline Cognition (r, p)

	Acute Plasticity		β-ERD Retention*		β-ERD Motor Preparation*	
	r	p	r	p	r	P
**Neurocognitive Composite**	0.20	0.386	**−0.60**	**0.005**	−0.36	0.121
**Auditory cognition (MCCB Verbal)**	**0.46**	**0.036**	**−0.55**	**0.012**	−0.21	0.368
**Attention and Vigilance**	0.16	0.482	**−0.62**	**0.004**	**−0.57**	**0.009**
**Working Memory**	0.21	0.369	**−0.60**	**0.005**	**−0.50**	**0.026**

* Spearman’s correlations used for β-ERD variables
